# Correction: Evaluation of a community-based HIV test and start program in a conflict affected rural area of Yambio County, South Sudan

**DOI:** 10.1371/journal.pone.0299906

**Published:** 2024-02-27

**Authors:** Cecilia Ferreyra, Laura Moretó-Planas, Fara Wagbo Temessadouno, Beatriz Alonso, Buai Tut, Victoria Achut, Mohamed Eltom, Endashaw M. Aderie, Vicente Descalzo-Jorro

There are errors in the Funding and Competing Interests statements. The correct statements are:

**Funding:** Médecins Sans Frontières (MSF) provided support in the form of salaries for CF, LMP, FWT, BA, BT, ME, EMA, and VDJ. The specific roles of these authors are articulated in the ‘author contributions’ section. All materials required for the study were paid by MSF as the sponsor of the research. The funder had no role in study design, data collection and analysis, decision to publish, or preparation of the manuscript.

**Competing Interests:** The authors have read the journal’s policy and have the following competing interests to declare: Médecins Sans Frontières (MSF) provided support in the form of salaries for CF, LMP, FWT, BA, BT, ME, EMA, and VDJ. This does not alter our adherence to *PLOS ONE* policies on sharing data and materials. There are no patents, products in development, or marketed products associated with this research to declare.
