# Supplementary material for: Evaluation of a community-based HIV test and start program in a conflict affected rural area of Yambio County, South Sudan
Source: PLoS One. 2021 Jul 12;16(7):e0254331. doi: 10.1371/journal.pone.0254331 (PMC8274874; doi:10.1371/journal.pone.0254331)
Supplement: Attachment — Submitted filename: Response to PLOS ONE Editors.docx [file pone.0254331.s001.docx]

Dear PLOS ONE Editors,

Thanks for accepting the publication of our manuscript entitled "Evaluation of a community-based HIV test and start program in a conflict affected rural area of Yambio County, South Sudan".  Please find below the answer to your recent queries.

1. Please include a separate legend for each figure in your manuscript.

CF: All legends for each figure have been included in the manuscript.

2. Thank you for clarifying your data access information. Based on the details you provided, we’ve drafted the following proposed Data Availability Statement for your review and approval:

“There are ethical restrictions on sharing the data as stated by both the MSF and South Sudan ERBs. Data cannot be shared without requesting permission, as defined in the MOU. The data underlying the results presented in the study are available from MSF OCBA. (www.msf.ocba) by contacting the medical director: cristian.casademont@barcelona.msf.org.”

Please confirm whether all information is accurate, and whether we may update your Data Availability Statement accordingly on your behalf.

CF: I confirm the above statement about data availability is accurate.

3. We note that you provided the guidelines from MSF here: <https://www.msf-me.org/photo-guidelines> and that you have not provided signed PLOS consent forms for the individuals who appear in Figures 1 and 2. Please note, if the participants did not complete this consent form, please remove this image from the figure, as we cannot publish the image without the signed PLOS Consent Form.

CF: as explained in a separate mail to the PLOS editorial team, we have tried to trace back the patients in order to get PLOS consent forms without any success. Instead we have changed the pictures (Fig 1 and 2) from MSF database to comply with PLOS guidelines.


Looking forward to hear about next steps.
Kind regards,

Dr Cecilia Ferreyra
