# Supplementary material for: Evaluation of a community-based HIV test and start program in a conflict affected rural area of Yambio County, South Sudan
Source: PLoS One. 2021 Jul 12;16(7):e0254331. doi: 10.1371/journal.pone.0254331 (PMC8274874; doi:10.1371/journal.pone.0254331)
Supplement: Attachment — Submitted filename: Review.docx [file pone.0254331.s002.docx]

Review

**Article**: PONE-D-20-06492R1
**Title**: Evaluation of a community-based HIV test and start program in a conflict affected rural

area of Yambio County, South Sudan

**Summary**: The study aims at improving HIV testing and ART coverage among people living in a conflict affected community of South Sudan in Africa. The investigators offered HIV counselling and testing in the study area and started on ART whoever tested HIV positive and was willing to do so. This is particularly an important study especially that it was conducted in a conflict affected community where access to HIV care may be challenging. The work in this manuscript is worth publishing and it fits very well within the UNAIDS strategy of 90-90-90 target of year 2020. However, I have some major and minor comments that need to be addressed before this work can be published.

**Abstract:**

Line 34, abbreviation for CAGs is presented for the first time before a full text is given.

Line 36, you mention “RIC was significantly higher at 18 months in patient under contingency plan (90% p<0.001) when compared to patients on regular follow up”. First, “patient” should be plural, i.e, “patients”. Also, you present one statistic in the comparison without giving the other one, which I find hanging. I suggest this be revised as follows “RIC was significantly higher at 18 months in patients under contingency plan when compared to patients on regular follow up (90% vs xx%, p<0.001)” where xx is RIC for patients on regular follow up.

In line 35-36, the 95% confidence interval is not appropriately presented, I would reorder to read as xx% [95% CI: xx-xx%]. This is the same issue even in the results section.

Under conclusion in line 40, the authors mention “Our study shows a high level of acceptance to HCT… “. This conclusion is not supported by the findings. It remains unclear how acceptance was measured, moreover, no summary finding about acceptance is presented in the abstract.

**Introduction:**

Lines 72-73, please provide references for the following statements, “… and ART coverage in the country is around 10%” and “An estimated 16000 new HIV infections happen every year and about…”

In line 103, a terminology “HIV testing and counselling (HTC)” is used yet under abstract in line 28 a statement “HIV counselling and testing (HCT)” is used. I suggest that for consistence, one terminology be used throughout.

**Methodology:**

In line 135, “All patient data was …”, data is plural. So, replace “was” with “were”. This needs to be observed throughout the manuscript. Also, in line 136, replace “where” with “were”. Note that Epidata is meant for data management not analysis. I would revise the statement “… paper forms were later on entered into Epidata database for further data management” (lines 136-137).

In line 141, you say the main outcome of interest was acceptability. I suppose you meant acceptability to HCT/HTC (please clarify). This being a main outcome, a clear methodology on how acceptability was measured and analysed should be presented. The focus seems to be on retention (as if it’s the main outcome) rather than acceptability.

In line 144, you mention “… factors associated with attrition”. While it’s true that attrition is a complement of retention, I would focus on analysing retention not attrition.

**Results:**

In line 237, a closing bracket in [IQR 262-620 is missing

In Table 1, a comma was used for % values instead of a decimal point. For example, under female category, the percentage is presented as 75,0 instead of 75.0. This is the same issue throughout Table 1.

In line 262, “loss” should be “lost”

In line 260, I would replace “Patient’s” with “Patients’ ”

In line 264, you present OR without first giving what this stands for. I suppose this stands for odds ratio. Also, no methodology presented on how mortality was analysed, for example, what statistical method was used to obtain the OR for mortality. Also, present the 95% CI for the OR throughout the results section.

In line 286, you say “a multivariate complete case Cox regression estimated that not being married (OR 2.5, p 0.001) …” Here it’s strange how you present OR from a Cox regression. Note that, Cox regression provides hazard ratio (HR) not OR. Please check your analysis methods. Also, please briefly describe how survival time was calculated, was any censoring done?

In line 268, “p 0.001” should be “p=0.001”. This is an issue almost everywhere in the manuscript. Please add the equal sign between p and the value throughout.

In line 279, the sentence “… were and 18 …” is not clear. I think some word is missing between “were” and “and”

In line 280, you present analysis on factors associated with detectable viral load, however, the methodology for analysing these factors is not described/mentioned in the methods section.

In Table 3, in the last row, “CI95%”, should be “95% CI”

In lines 291-292, you say “At 12 months, 4 were LTFU, no death, 65 still in follow up”. This adds up to 69. Were all 72 available at 12 months? If yes, what happened to the 3 patients?

In line 294, you say “RIC was significantly higher (90% at 18 months, p<0.001) when compared to patients on regular follow up…”. It’s not clear what group of patients the statement refers to. Also, present RIC for patients on regular follow up and the test statistic used to compare the two.

In Fig 6 (as well as Fig 4 and Fig 7), I suggest you revise the y-axis label to "Proportion retained in care" drop % since the y-axis is showing the proportion not percentage. Also, in Fig 6, use p<0.001 (not p-value<0.001) for consistence, x-axis should indicate unit of measurement for analysis time (I think months), let one line be dotted or long-dashed for readable print out (black and white), please add number at risk (also for Fig 4 and Fig 7).

In line 304, I think “p 0.014” is showing the overall difference in retention but not the difference at 12 or 18 months. Please clarify and revise accordingly. Also, it's not clear what test statistic was used for this comparison.

Lines 308-330, if acceptability is the main outcome as mentioned in the methodology, then I would present this before retention.

Line 309, it’s not clear how the number 279 was determined

Line 315, “a” should be “at”

Fig 5 is faint and of low quality. The label on third bar is cut off. Since the figure shows both numbers and percentages, I suggest a two-y-axis figure, one axis showing numbers and the other one showing the percentage.

**Discussion:**

Line 332, you say “our study shows a high level of acceptance to HCT…”, I am struggling to see how this is shown by the study findings. What result are you basing on to claim this?

Line 355-365, you say “Patients characteristics associated with better adherence …”, I did not see any analysis and results on adherence before this statement. Also, it's not mentioned how adherence was measured. Please clarify.

**Conclusion:**

Lines 367-372, again, what was the main outcome of this study? In the methodology, line 141, the main outcome is acceptability, but no conclusion is presented on this.

Lines 371-372, you say “Our study shows that differentiated models of care are feasible in these contexts”. I find this conclusion inappropriate since it is not supported by your findings. The study did not study the feasibility of differentiated models but rather community HCT and ART initiation.
