# Supplementary material for: Evaluation of a community-based HIV test and start program in a conflict affected rural area of Yambio County, South Sudan
Source: PLoS One. 2021 Jul 12;16(7):e0254331. doi: 10.1371/journal.pone.0254331 (PMC8274874; doi:10.1371/journal.pone.0254331)
Supplement: Attachment — Submitted filename: Yambio Rebuttal Letter MAY2021.DOCX [file pone.0254331.s003.DOCX]

Dear PLOS ONE Editors,

**Article**: PONE-D-20-06492R1
**Title**: Evaluation of a community-based HIV test and start program in a conflict affected rural

area of Yambio County, South Sudan

Thanks for accepting the publication of our manuscript entitled "Evaluation of a community-based HIV test and start program in a conflict affected rural area of Yambio County, South Sudan".  Please find below the answer to your recent queries.

---------------------------------------------------------------------------------------------------------------------------

**Summary**: The study aims at improving HIV testing and ART coverage among people living in a conflict affected community of South Sudan in Africa. The investigators offered HIV counselling and testing in the study area and started on ART whoever tested HIV positive and was willing to do so. This is particularly an important study especially that it was conducted in a conflict affected community where access to HIV care may be challenging. The work in this manuscript is worth publishing and it fits very well within the UNAIDS strategy of 90-90-90 target of year 2020. However, I have some major and minor comments that need to be addressed before this work can be published.

**Abstract:**

Line 34, abbreviation for CAGs is presented for the first time before a full text is given.

CF: the term is now written in full and abbreviation put under bracket,

Line 36, you mention “RIC was significantly higher at 18 months in patient under contingency plan (90% p<0.001) when compared to patients on regular follow up”. First, “patient” should be plural, i.e, “patients”. Also, you present one statistic in the comparison without giving the other one, which I find hanging. I suggest this be revised as follows “RIC was significantly higher at 18 months in patients under contingency plan when compared to patients on regular follow up (90% vs xx%, p<0.001)” where xx is RIC for patients on regular follow up.

CF: the word “patient has been changed to “patients” in line 36. Regarding your comment on RIC, the sentence has been modified as follow in line 40: RIC was significantly higher at 18 months in patients under CAGs when compared to patients on regular follow up (90.9% vs 63.4%, p,0.001)”

In line 35-36, the 95% confidence interval is not appropriately presented, I would reorder to read as xx% [95% CI: xx-xx%]. This is the same issue even in the results section.

CF: the 95% CI is now presented as suggested in both the abstract and the results sections.

Under conclusion in line 40, the authors mention “Our study shows a high level of acceptance to HCT… “. This conclusion is not supported by the findings. It remains unclear how acceptance was measured, moreover, no summary finding about acceptance is presented in the abstract.

CF: thanks for the comment, we have added a line in the abstract about the acceptability part and then explained further in methodology and results, also the sentence about acceptance has been modified as follows “Our study shows that HCT and early ART initiation in conflict affected populations can be provided with good program outocomes”..

**Introduction:**

Lines 72-73, please provide references for the following statements, “… and ART coverage in the country is around 10%” and “An estimated 16000 new HIV infections happen every year and about…”

CF: the ART coverage and number of new infections have been updated and the following references have been included:

- Jervase A et all, UNAIDS report 2018 and UNHCR South Sudan – Conflict, displacement, famine and the HIV response, Time to act!

- UNAIDS. HIV/AIDS, South Sudan, 2019 [Internet].

In line 103, a terminology “HIV testing and counselling (HTC)” is used yet under abstract in line 28 a statement “HIV counselling and testing (HCT)” is used. I suggest that for consistence, one terminology be used throughout.

CF: we have now kept it consistent as HIV counseling and testing (HCT) throughout the manuscript.

**Methodology:**

In line 135, “All patient data was …”, data is plural. So, replace “was” with “were”. This needs to be observed throughout the manuscript. Also, in line 136, replace “where” with “were”. Note that Epidata is meant for data management not analysis. I would revise the statement “… paper forms were later on entered into Epidata database for further data management” (lines 136-137).

CF: “all patients data” sentence has been changed to plural and revised throughout the manuscript. The Epidata usage is now appropriately mentioned as *for data management.*

In line 141, you say the main outcome of interest was acceptability. I suppose you meant acceptability to HCT/HTC (please clarify). This being a main outcome, a clear methodology on how acceptability was measured and analysed should be presented. The focus seems to be on retention (as if it’s the main outcome) rather than acceptability.

CF: the aim of the study was to assess feasibility of community-based HIV care in this conflict affected population, which was measured through programme outcomes (RIC, LFU, viral suppression and mortality). A qualitative component of the programme included programme acceptability interviews in the community and direct beneficiaries of the programme. We have modified this paragraph as follow: “the aim of the study was to describe programme outcomes which included retention in care, viral suppression, LFU and death at 12 and 18 months of programme initiation.”

As per the acceptability part, we modified the paragraph in line 148 as follow: “A semi-qualitative assessment using questionnaires was done at community level to assess acceptability, including participants as follows:…”

In line 144, you mention “… factors associated with attrition”. While it’s true that attrition is a complement of retention, I would focus on analysing retention not attrition.

CF: the sentence has been modified to: “…factors associated with RIC.”

**Results:**

In line 237, a closing bracket in [IQR 262-620 is missing

CF: corrected, bracket closed.

In Table 1, a comma was used for % values instead of a decimal point. For example, under female category, the percentage is presented as 75,0 instead of 75.0. This is the same issue throughout Table 1.

CF: all commas in Table 1 where change to decimals.

In line 262, “loss” should be “lost”

CF: now corrected as LFU

In line 260, I would replace “Patient’s” with “Patients’ ”

CF: this is now corrected to “Patients’”

In line 264, you present OR without first giving what this stands for. I suppose this stands for odds ratio. Also, no methodology presented on how mortality was analysed, for example, what statistical method was used to obtain the OR for mortality. Also, present the 95% CI for the OR throughout the results section.

CF: the mortality analysis was not found to be relevant for the discussion, hence the sentence has been deleted to kept consistency in reporting other programme outcomes (RIC, viral suppression and LFU) as suggested by the reviewers.

In line 286, you say “a multivariate complete case Cox regression estimated that not being married (OR 2.5, p0.001) …” Here it’s strange how you present OR from a Cox regression. Note that, Cox regression provides hazard ratio (HR) not OR. Please check your analysis methods. Also, please briefly describe how survival time was calculated, was any censoring done?

CF: thanks for highlighting this error. Indeed, we calculated Hazard Ratios (HA) and not Odds Ratios (OR). The analysis method has been clarified and the sentence has been modified in line 274.

In line 268, “p 0.001” should be “p=0.001”. This is an issue almost everywhere in the manuscript. Please add the equal sign between p and the value throughout.

CF: this is now corrected throughout to mean “p=0.0x”

In line 279, the sentence “… were and 18 …” is not clear. I think some word is missing between “were” and “and”

CF: this is now corrected; it was a mistake from the previous version.

In line 280, you present analysis on factors associated with detectable viral load, however, the methodology for analysing these factors is not described/mentioned in the methods section.

CF: a sentence to clarify the method to analyse viral load has been included in methods in line 149 as follow: “We used a stepwise backward regression technique at p-value<0.2 to include variables in risk factor assessment model. All variables with p-value >0,2 were sequentially removed in the process; a p-value <0.05 was used for inclusion of factors in the final model”

In Table 3, in the last row, “CI95%”, should be “95% CI”

CF: corrected.

In lines 291-292, you say “At 12 months, 4 were LTFU, no death, 65 still in follow up”. This adds up to 69. Were all 72 available at 12 months? If yes, what happened to the 3 patients?

CF: the 3 patients were handed over to other program during the period, which is now included in the text in line 298.

In line 294, you say “RIC was significantly higher (90% at 18 months, p<0.001) when compared to patients on regular follow up…”. It’s not clear what group of patients the statement refers to. Also, present RIC for patients on regular follow up and the test statistic used to compare the two.

CF: we are comparing RIC in CAGs and non-CAGs/”patients under regular follow up” with the mobile teams and included the statistics to compare both. Kaplan Meir was used to measure RIC. The sentence has been modified as follows: “RIC in patients on CAG was significantly higher (90.9% vs 63.4%, p <0.001) than patients under regular follow up by the mobile teams (non-GAG) “.

In Fig 6 (as well as Fig 4 and Fig 7), I suggest you revise the y-axis label to "Proportion retained in care" drop % since the y-axis is showing the proportion not percentage. Also, in Fig 6, use p<0.001 (not p-value<0.001) for consistence, x-axis should indicate unit of measurement for analysis time (I think months), let one line be dotted or long-dashed for readable print out (black and white), please add number at risk (also for Fig 4 and Fig 7).

CF: This is now corrected, and improved as per the suggestions in Fig 4, 6 and 7..

In line 304, I think “p 0.014” is showing the overall difference in retention but not the difference at 12 or 18 months. Please clarify and revise accordingly. Also, it's not clear what test statistic was used for this comparison.

CF: thanks for the remark, indeed the figure is showing overall RIC and not the difference, this has now been clarified in the manuscript as follow: “Kaplan-Meier estimated that overall RIC of these patients was 87.8% [95% CI: 79.5-92.9] ,which was significantly higher than for the regular cohort followed up by the mobile teams (p=0.014). The statistic test used for the comparison was logrank test, and this has been updated in the text accordingly.

Lines 308-330, if acceptability is the main outcome as mentioned in the methodology, then I would present this before retention.

CF: as clarified before the main outcomes of the study were those related with RIC. We have included a paragraph in the methodology section, line 235, to better explain the acceptability component.

Line 309, it’s not clear how the number 279 was determined

CF: a sample size of 341 participants was initiatly estimated, due to the security constrains for the area the team managed to perform 279 (82%) of the interviews. This has been now included in line 334-336.

Line 315, “a” should be “at”

CF: thanks for the remark, it has now been changed.

Fig 5 is faint and of low quality. The label on third bar is cut off. Since the figure shows both numbers and percentages, I suggest a two-y-axis figure, one axis showing numbers and the other one showing the percentage.

CF: figures have been revised as suggested above.

**Discussion:**

Line 332, you say “our study shows a high level of acceptance to HCT…”, I am struggling to see how this is shown by the study findings. What result are you basing on to claim this?

CF: as suggested above we have modified the the main outcomes as follows: “our study shows RIC and VL suppression results similar to those observed in stable contexts and with HIV services provided at a health care facility”.

Line 355-365, you say “Patients characteristics associated with better adherence …”, I did not see any analysis and results on adherence before this statement. Also, it's not mentioned how adherence was measured. Please clarify.

CF: thanks for the comment. We have remove the sentence to avoid confusions.

**Conclusion:**

Lines 367-372, again, what was the main outcome of this study? In the methodology, line 141, the main outcome is acceptability, but no conclusion is presented on this.

CF: as mentioned above the main outcome was RIC and viral suppression, appart from death and LFU. This has been modified along the manuscript.

Lines 371-372, you say “Our study shows that differentiated models of care are feasible in these contexts”. I find this conclusion inappropriate since it is not supported by your findings. The study did not study the feasibility of differentiated models but rather community HCT and ART initiation.

CF: Thanks for your comment, we have modified the sentence to clearly indicate the main purpose of the study as follows: “Our study shows that provision of HIV services at community level can be done with good programme outcomes suggesting that it could be replicated in similar settings to ensure PLWHIs are included in the health response in conflict affected settings”.
